# Effects of esketamine–sufentanil for patient-controlled intravenous analgesia in women following cesarean section: A randomized clinical trial

**DOI:** 10.3389/fphar.2025.1579633

**Published:** 2025-04-14

**Authors:** Yanping Zhao, Lin Liu, Yi Hou, Jiaming Fan

**Affiliations:** ^1^ Department of Anesthesiology, Women and Children’s Hospital of Jiaxing University, Jiaxing, China; ^2^ Department of Obstetrics, Women and Children’s Hospital of Jiaxing University, Jiaxing, China

**Keywords:** esketamine, sufentanil, cesarean section, postoperative analgesia, nausea and vomiting

## Abstract

**Background:**

Postoperative pain following cesarean section can cause maternal anxiety, limited ambulation, and even postpartum depression. In this study, we aimed to investigate the effects of esketamine for postoperative patient-controlled intravenous analgesia in women following cesarean section.

**Methods:**

One hundred women were randomly assigned to two groups. The esketamine group received 1 mg⋅kg^-1^⋅d^-1^ of esketamine +1 µg⋅kg^-1^⋅d^-1^ of sufentanil for intravenous postoperative analgesia, and the control group received 1 µg⋅kg^-1^⋅d^-1^ of sufentanil for intravenous analgesia. The primary outcome was the pain intensity during the postoperative 24 h, and it was assessed using a visual analog scale (VAS). The secondary outcomes included hemodynamic parameters, total consumption of analgesics, blood loss, and drug-related side effects (hypotension, hypertension, bradycardia, nausea, and vomiting).

**Results:**

The VAS scores at rest were lower in the esketamine group than in the control group during the postoperative 6 h–24 h (*p* < 0.05), and the VAS scores at cough in the esketamine group were lower during the postoperative 4 h–24 h (*p* < 0.05). There were significant differences at blood loss during the postoperative 24 h (137.6 ± 33.0 vs 159.6 ± 41.3 mL, *p* = 0.004). Blood pressure and heart rate were greater in the esketamine group than in the control group during the postoperative 8 h–24 h (*p* < 0.05). The incidence of nausea and vomiting was significantly lower in the esketamine group than in the control group (4% vs 18%, *p* = 0.025).

**Conclusion:**

This study indicated that esketamine not only improved postoperative pain but also reduced postpartum blood loss and the incidence of nausea and vomiting in women undergoing cesarean section (registration number: ChiCTR2400082094).

**Systematic Review Registration:**

https://www.chictr.org.cn, Identifier ChiCTR2400082094

## 1 Introduction

Postoperative pain after a cesarean section can cause maternal irritability, limit ambulation, impair postoperative recovery, and even lead to postpartum depression (Gamez and Habib, 2018). Esketamine is the dextrorotatory form of ketamine, which has a higher affinity for N-methyl-D-aspartate (NMDA) receptors and μ-opioid receptors than ketamine; therefore, it has a more potent analgesic effect (van de Bunt et al., 2017). The therapeutic dose of esketamine is only half that of ketamine, with a higher clearance rate and a lower incidence of side effects (Wang et al., 2019). Previous studies on esketamine have focused on the prevention of postpartum depression (Daly et al., 2019; Rodolico et al., 2024; Niu et al., 2024). The literature reported that ketamine could enhance uterine contraction (Gertie et al., 1979). However, there are fewer studies on esketamine for postoperative intravenous analgesia and postpartum blood loss following cesarean section. Therefore, in this study, we investigated the effects of esketamine–sufentanil for patient-controlled intravenous analgesia in women undergoing caesarean section.

## 2 Materials and methods

### 2.1 Study population

This study was approved by the hospital Ethics Committee (approval number: 2023-KY002), and informed consent was obtained from the patients. This study was registered in the Chinese Clinical Trial Registry (registration number: ChiCTR2400082094) on 20 March 2024. A total of 100 women underwent cesarean section between May 2024 and November 2024. The inclusion criteria included the following: ASA level 1–2, age 20–35 years, gestational age 37–41 weeks, and weight 55–85 kg. The exclusion criteria included the following: patients with pregnancy-related hypertension, nervous system disease, coagulation dysfunction, placenta implantation, and placenta previa. According to a numerical randomization table, 100 women were divided into two groups, with 50 cases in each group. Randomization was carried out by opening a sealed envelope containing a serial number. The allocation sequence was generated using random permuted block randomization. The investigators, anesthesiologists, and surgeons were blinded to this study. Study drugs were prepared by an anesthesiologist who was blinded to the allocation.

All women were not given preoperative medications. After admission to the room, intravenous access was established, and Ringer’s lactate solution was infused with 8–10 mL⋅kg^-1^⋅h^-1^; electrocardiogram, blood pressure, pulse oximetry (SpO_2_), and heart rate (HR) were routinely monitored. After a successful anesthesia puncture, 10 mg of hyperbaric bupivacaine 2 mL was injected into the subarachnoid space over 10 s with the needle orifice facing cephalad; then, patients were immediately turned to the supine position. After delivery of the fetus, 5 U of intravenous oxytocin was administered to enhance uterine contraction. A surgical incision was permitted after bilateral T6 sensory block of pain was achieved. At the end of the operation, the esketamine group was given 1 mg⋅kg^-1^⋅d^-1^ esketamine (Jiangsu Hengrui Pharmaceutical Company, China) + 1 μg⋅kg^-1^⋅d^-1^ sufentanil (Yichang Renfu Pharmaceutical Company, China) + normal saline diluted to 100 mL for postoperative patient-controlled intravenous analgesia, and the control group used 1 μg⋅kg^-1^⋅d^-1^ sufentanil + saline diluted to 100 mL for postoperative patient-controlled intravenous analgesia. The parameters of the analgesic pump (Sujia Medical Devices Co., Ltd.) were set as follows: background infusion dose was 2 mL/h, bonus dose was 2 mL, and locking time was 15 min. All procedures were completed under spinal anesthesia in both groups.

### 2.2 Measurement

Maternal blood pressure and heart rate were measured at 1-h intervals within the postoperative 24 h. The maternal pain intensity was assessed using the visual analog scale (VAS: 0 = no pain, 1–3 = mild pain, 4–7 = moderate pain, and 8–10 = severe pain) at rest and cough within 24 h postoperatively, and the postoperative drug-related complications were recorded. Hypertension was defined as systolic blood pressure greater than 120% of baseline, hypotension was defined as systolic blood pressure less than 80% of baseline, and bradycardia was defined as the heart rate less than 60 beats/min. Ephedrine 6 mg was administered intravenously when systolic blood pressure was lower than 80% of baseline, and atropine 0.25–0.5 mg was administered intravenously when the postoperative heart rate was lower than 50 beats/min. Respiratory depression was defined as pulse oximetry less than 94% on air inhalation. The occurrence of postoperative complications such as maternal hypertension, hypotension, tachycardia, bradycardia, nightmares, respiratory depression, nausea, and vomiting was recorded. Postpartum blood loss was calculated using the weighing method (1,000 g ≈ 1,000 mL).

### 2.3 Sample size

The primary outcome of this trial was the pain intensity; the second outcomes were the drug-related side effects. Based on our pilot study (with 10 participants in each group), the VAS scores at 4 h postoperatively were 3.2 ± 0.8 in the esketamine group and 3.7 ± 0.8 in the control group. We calculated that 41 patients in each group were required to detect a statistical difference in VAS scores between the two groups, with an α of 0.05 and a power of 0.80. The sample size was increased to 50 to allow for any dropout in each group.

### 2.4 Statistics

Data analysis was performed using the SPSS 20.0 statistical software package, version 20.0 (SPSS Inc., Chicago, IL). Numerical data with a normal distribution were presented as mean ± standard deviation and analyzed with a *t*-test and two-way analysis of variance. Numerical data with a non-normal distribution were shown as median (interquartile range, IQR), and we used the Mann–Whitney U-test for analysis. Categorical variables were presented as numbers (%) and analyzed with a χ^2^ or Fisher’s exact test. It was considered statistically significant as *p* < 0.05.

## 3 Results

**FIGURE 1:**
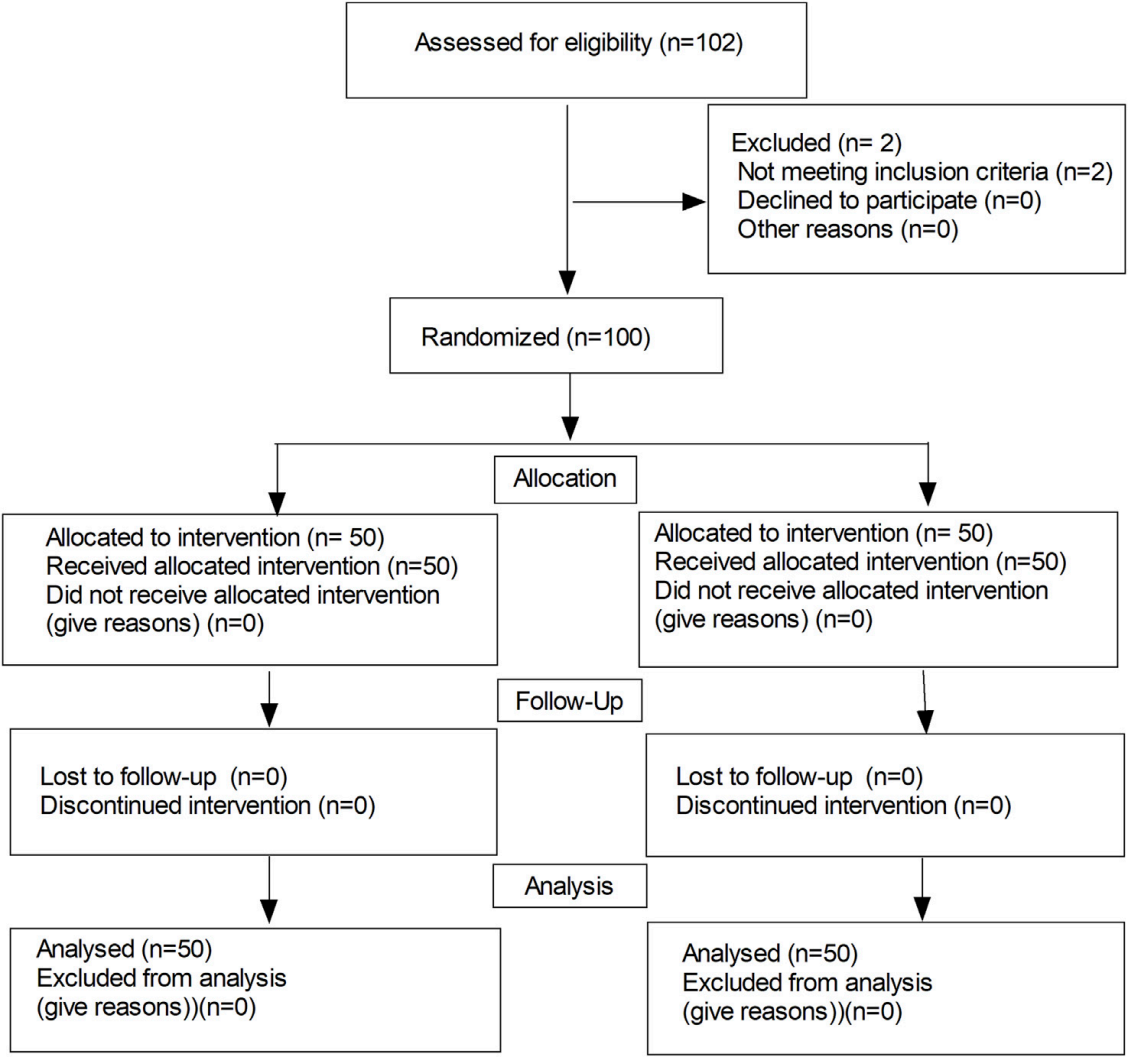
Flow diagram of the study.

The flow diagram of the study is shown in Figure 1. One-hundred and two patients were recruited, and then 100 subjects were randomly categorized into two groups. There were no significant differences in the maternal age, weight, height, and gestational week between the two groups (Table 1). There were significant differences in terms of the total consumption of analgesics and blood loss during the postoperative 24 h (56.8 ± 4.3 vs 58.9 ± 4.8 mL, *p* = 0.022; 137.6 ± 33.0 vs 159.6 ± 41.3 mL, *p* = 0.004, respectively). Meanwhile, no differences were observed in terms of the duration of operation, the highest levels of sensory block, the total amount of fluid, use of ephedrine, use of atropine, and the total blood loss within the postoperative 24 h (in Table 2, *p* > 0.05).

**TABLE 1:** Characteristics of the parturient.

Index	Esketamine group (*n* = 50)	Control group (*n* = 50)	*P*-value
Age (year)	31.0 ± 3.9	30.6 ± 4.7	0.963
Height (cm)	160.6 ± 4.6	161.3 ± 4.2	0.440
Weight (kg)	69.9 ± 8.2	71.9 ± 8.4	0.225
Gestational age (week)	38.7 ± 0.8	38.4 ± 0.9	0.136

Data are presented as mean ± SD.

**TABLE 2:** Maternal outcomes.

Index	Esketamine group (*n* = 50)	Control group (*n* = 50)	*P*-value
Duration of operation (min)	46.6 ± 5.9	45.3 ± 5.3	0.239
Highest levels of sensory block [T4/T6] (n)	16/34	17/33	0.679
Amount of fluid during the operation (mL)	719.4 ± 47.1	727.8 ± 84.9	0.542
Use of ephedrine (n)	32	33	0.834
Use of atropine (n)	0	1	0.610
Total consumption of analgesics during the 24 h (mL)	56.8 ± 4.3	58.9 ± 4.8	0.022*
Blood loss during the postoperative 24 h (mL)	137.6 ± 33.0	159.6 ± 41.3	0.004^*^

Data are presented as mean ± SD or number.

*P < 0.05.

**FIGURE 2:**
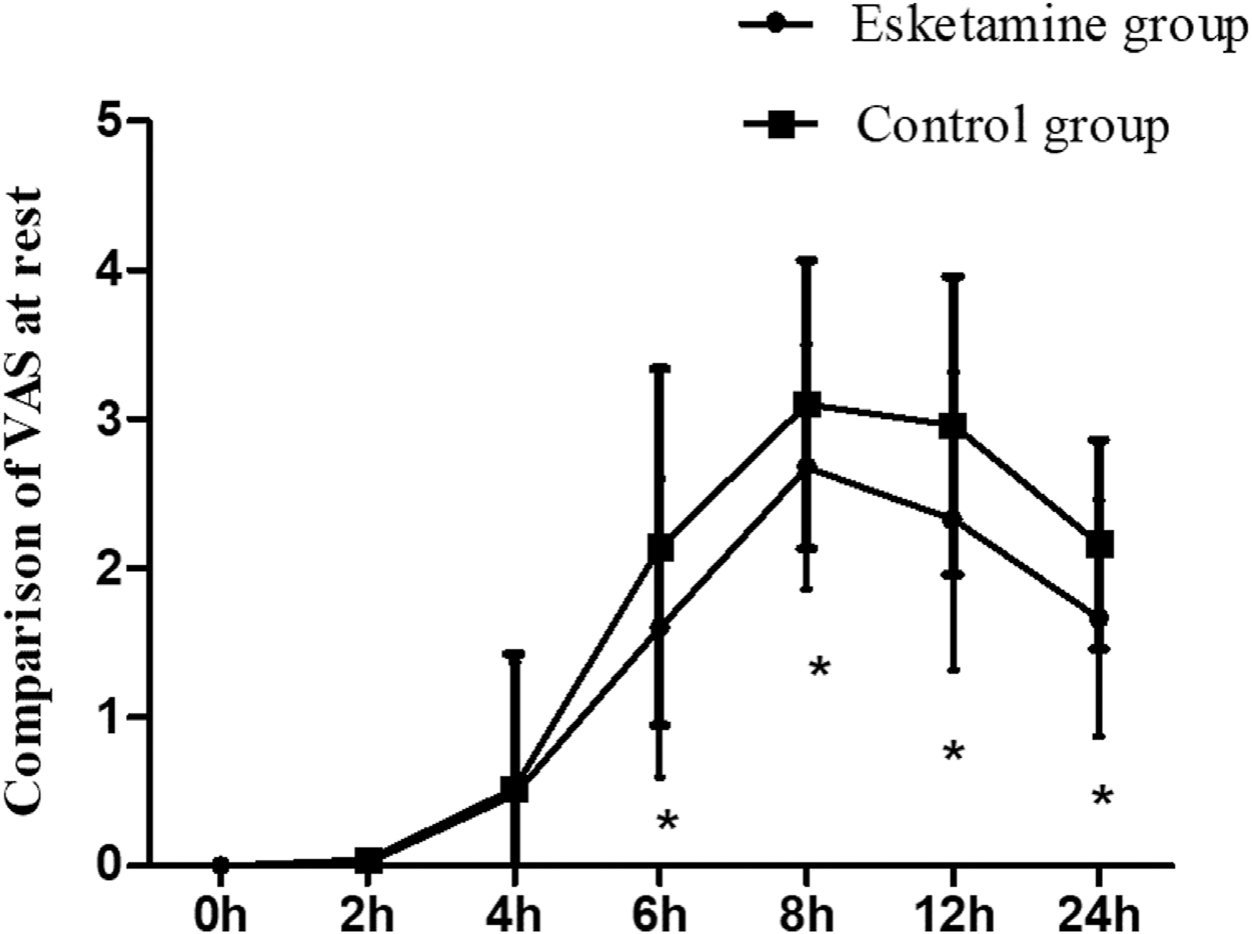
Comparison of VAS scores at rest between the two groups, *p < 0.05.

**FIGURE 3:**
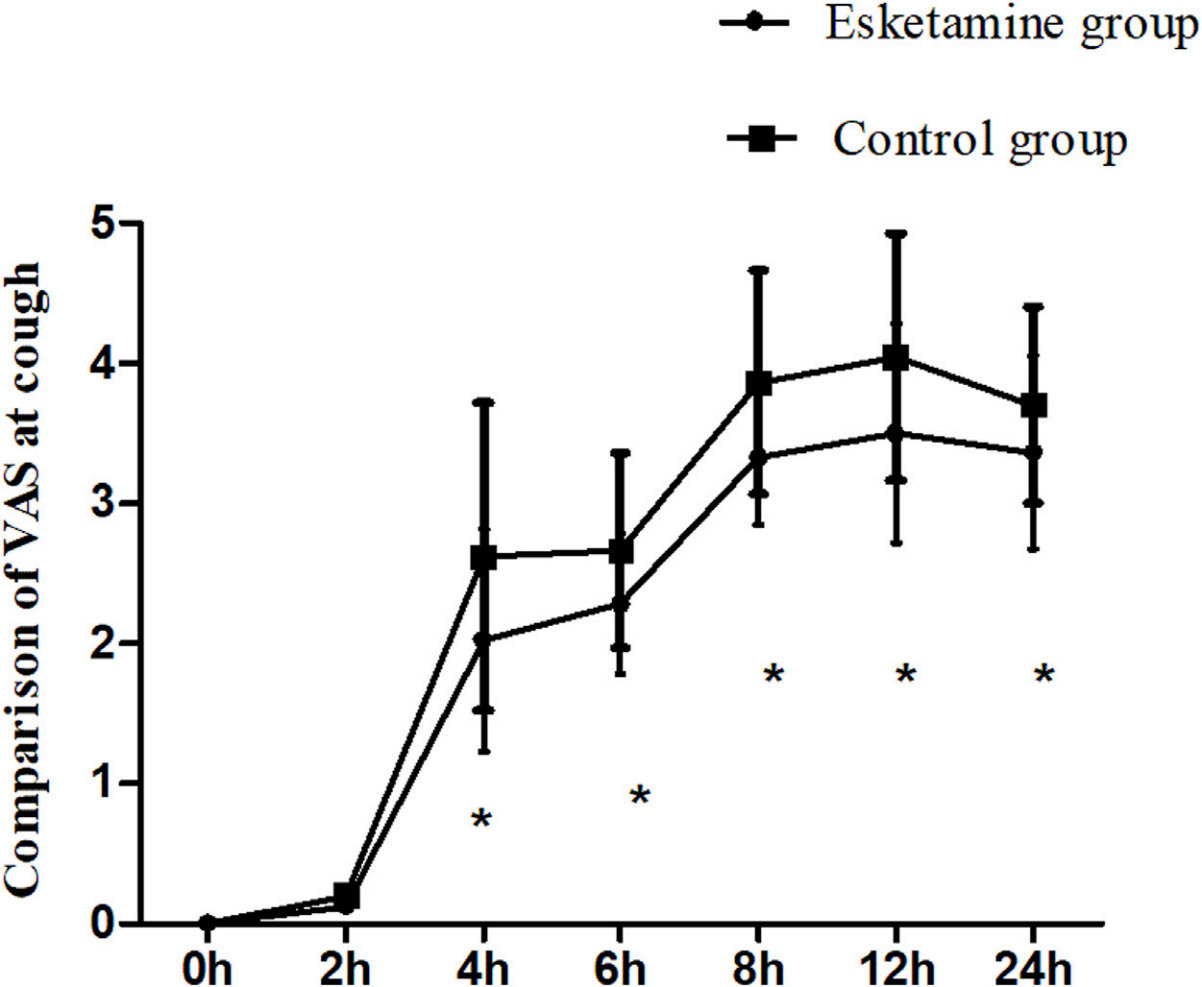
Comparison of VAS scores at cough between the two groups, *p < 0.05.

The VAS scores at rest were lower in the esketamine group than in the control group during the postoperative 6 h–24 h (*p* < 0.05). However, there were no significant differences at other time points between the two groups (Figure 2). The VAS scores at cough in the esketamine group were lower during the postoperative 4 h–24 h (*p* < 0.05, in Figure 3).

**FIGURE 4:**
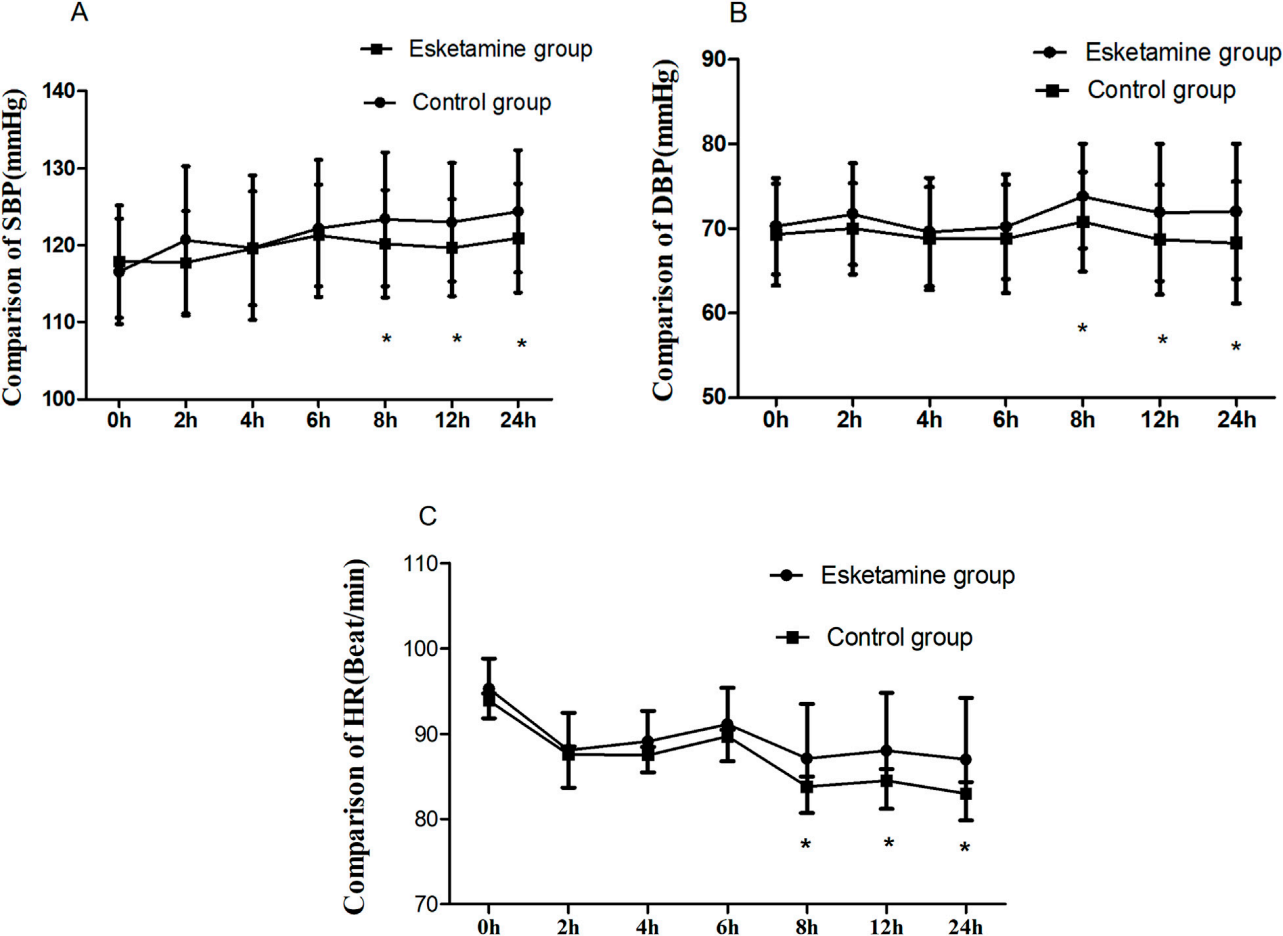
Comparison of SBP, DBP, and HR between the two groups, *p < 0.05.

Blood pressure and HR were greater in the esketamine group than in the control group during the postoperative 8 h–24 h (*p* < 0.05). Meanwhile, the SBP, DBP, and HR were similar within the first postoperative 6 h, and there were no significant differences between the two groups (Figure 4).

The incidence of nausea and vomiting was significantly lower in the esketamine group than in the control group (4% vs 18%, *p* = 0.025), but there were no statistical significances in terms of hypotension, hypertension, respiratory depression, tachycardia, bradycardia, and nightmare between the two groups (*p* > 0.05) (Table 3).

**TABLE 3:** Adverse events of parturient (n = 50).

Index	Esketamine group	Control group	*P-*value
Hypotension (n)	1	2	0.999
Respiratory depression (n)	0	0	—
Hypertension (n)	1	0	0.610
Tachycardia (n)	1	0	0.610
Bradycardia (n)	0	0	—
Nightmare (n)	2	0	0.475
Nausea and vomiting (n)	2	9	0.025*

Data are expressed as numbers.

*P < 0.05.

## 4 Discussion

In this study, we found that esketamine not only improved postoperative pain but also reduced postpartum blood loss and the incidence of nausea and vomiting in women undergoing cesarean section.

Esketamine is an intravenous anesthetic, which produces analgesic effects by blocking NMDA receptors (van de Bunt et al., 2017). Sufentanil is an opioid drug that may produce analgesic effects mainly by activating the μ_1_ receptor. Compared with opioid drugs, esketamine has fewer effects on respiratory depression (Brinck et al., 2021). In this study, the VAS score was significantly lower in the esketamine group than in the control group during postoperative 4–24 h, and the total consumption of analgesics was decreased, indicating that esketamine could enhance the postoperative analgesic effect of sufentanil and can effectively alleviate the postoperative pain in women undergoing cesarean section. Chen Y. et al. (2024) found that esketamine, as an adjuvant in patient-controlled intravenous analgesia, decreased the postoperative pain scores in patients undergoing elective cesarean section. Li et al. (2024) also found that esketamine decreased sufentanil consumption and pain scores at movement when esketamine was used for the treatment of postpartum depression following cesarean section. Esketamine with sufentanil was used for patient-controlled intravenous analgesia in women undergoing cesarean section; it could provide a better postoperative analgesic effect, which was associated with blocking NMDA receptors and activating μ opioid receptors.

Esketamine has an excitatory effect on the cardiovascular system, which may cause an increase in blood pressure and heart rate (Del Sant et al., 2020; Wang Q. et al., 2022). In our study, the postoperative blood pressure and HR were similar between the two groups within the first 6 h postoperatively. Meanwhile, the postoperative blood pressure and HR were greater in the esketamine group than in the control group during the postoperative 8 h–24 h. This might be related to the total dose of esketamine. When esketamine was continuously administered by intravenous infusion, the dose of esketamine was insufficient to cause a significant excitation of the cardiovascular system or significant increases in blood pressure and heart rate within the first 6 h postoperatively. Furthermore, esketamine could alleviate the sympathetic nerve block-induced decrease of blood pressure and heart rate after spinal anesthesia, thus maintaining hemodynamic stability after surgery. On the other hand, postoperative pain following cesarean section would lead to an increase in blood pressure and heart rate. Esketamine may attenuate the sympathetic nerve block-induced fall in blood pressure and heart rate during spinal anesthesia, thus maintaining hemodynamic stability. It has a certain preventive effect on spinal anesthesia-induced hypotension.

Reducing postpartum bleeding has a positive impact on postoperative recovery. In our study, the amount of blood loss during the postoperative 24 h was reduced in the esketamine group, mainly because esketamine caused peripheral vasoconstriction and uterine contractions. Xiang et al. (2020) found that topical ketamine caused peripheral vasoconstriction following hemorrhage in rats, but fentanyl induced vasodilation. Moreover, ketamine had no effect on compensatory vasoconstriction after hemorrhage. However, following fentanyl, arteriolar constriction disappeared and was replaced by persistent vasodilation. In this study, blood loss during the postoperative 24 h was reduced by 22 mL; it was of little clinical significance. However, it had a certain reference value and guiding significance for clinical practice, and provided a new direction for preventing postpartum bleeding. The mechanism of action of esketamine in reducing postoperative blood loss needs further investigation.

In the present study, we also found that esketamine could reduce the incidence of nausea and vomiting. However, there was no significant differences in the incidence of maternal hypertension, tachycardia, and psychiatric symptoms. Esketamine has a certain sympathetic excitatory effect, reducing hypoperfusion stimulation of the emetic center caused by nausea and vomiting. Moreover, due to sympathetic excitation, vagal activity and gastrointestinal activity are weakened, thus reducing the incidence of nausea and vomiting. The literature reported that esketamine decreased postoperative nausea and vomiting in patients undergoing elective cesarean section (Wang W. et al., 2022). It was similar to our results. Esketamine did not increase the incidence of drug-related adverse reactions in our study. The use of esketamine during cesarean section is said to be a double-edged sword. Recently, research has suggested that esketamine used for postoperative pain management can lead to improvements in postpartum depression and pain outcomes (Chen HZ. et al., 2024; Jiang and Xu, 2024; Wen et al., 2024). Meanwhile, esketamine increases the risk of psychiatric symptoms (Shoib et al., 2022). It is necessary to consider the advantages and disadvantages of pain control with esketamine during cesarean section, as well as the risk of it inducing psychiatric symptoms.

## 5 Limitations

Individual differences in pain may influence the results. Moreover, the sample size is small, which will affect the accuracy of research results. Finally, the safety of esketamine in the neonates needs further research.

## 6 Conclusion

This study indicated that esketamine not only improved postoperative pain but also reduced postpartum blood loss and the incidence of nausea and vomiting in women undergoing cesarean section.

## Data Availability

The raw data supporting the conclusions of this article will be made available by the authors, without undue reservation.
